# Cysteine Cathepsins Inhibition Affects Their Expression and Human Renal Cancer Cell Phenotype

**DOI:** 10.3390/cancers12051310

**Published:** 2020-05-21

**Authors:** Magdalena Rudzińska, Alessandro Parodi, Valentina D. Maslova, Yuri M. Efremov, Neonila V. Gorokhovets, Vladimir A. Makarov, Vasily A. Popkov, Andrey V. Golovin, Evgeni Y. Zernii, Andrey A. Zamyatnin

**Affiliations:** 1 Institute of Molecular Medicine, Sechenov First Moscow State Medical University, 119991 Moscow, Russia; magdda.rudzinska@gmail.com (M.R.); aparodi.sechenovuniversity@gmail.com (A.P.); gorokhovets@gmail.com (N.V.G.); known.sir@yandex.ru (V.A.M.); golovin.andrey@gmail.com (A.V.G.); zerni@belozersky.msu.ru (E.Y.Z.); 2 Faculty of Bioengineering and Bioinformatics, Moscow State University, 119992 Moscow, Russia; valmarfgecnf@gmail.com; 3 Institute for Regenerative Medicine, Sechenov University, 119991 Moscow, Russia; yu.efremov@gmail.com; 4 Belozersky Institute of Physico-Chemical Biology, Lomonosov Moscow State University, 119992 Moscow, Russia; popkov.vas@gmail.com; 5 Shemyakin-Ovchinnikov Institute of Bioorganic Chemistry, Russian Academy of Sciences, 117997 Moscow, Russia

**Keywords:** cysteine cathepsins, cysteine cathepsin inhibitors, lysosome, renal cancer

## Abstract

Renal cancer would greatly benefit from new therapeutic strategies since, in advanced stages, it is refractory to classical chemotherapeutic approaches. In this context, lysosomal protease cysteine cathepsins may represent new pharmacological targets. In renal cancer, they are characterized by a higher expression, and they were shown to play a role in its aggressiveness and spreading. Traditional studies in the field were focused on understanding the therapeutic potentialities of cysteine cathepsin inhibition, while the direct impact of such therapeutics on the expression of these enzymes was often overlooked. In this work, we engineered two fluoromethyl ketone-based peptides with inhibitory activity against cathepsins to evaluate their potential anticancer activity and impact on the lysosomal compartment in human renal cancer. Molecular modeling and biochemical assays confirmed the inhibitory properties of the peptides against cysteine cathepsin B and L. Different cell biology experiments demonstrated that the peptides could affect renal cancer cell migration and organization in colonies and spheroids, while increasing their adhesion to biological substrates. Finally, these peptide inhibitors modulated the expression of LAMP1, enhanced the expression of E-cadherin, and altered cathepsin expression. In conclusion, the inhibition of cysteine cathepsins by the peptides was beneficial in terms of cancer aggressiveness; however, they could affect the overall expression of these proteases.

## 1. Introduction

Cysteine cathepsins (Cts) are lysosomal proteases belonging to the C1 family of papain-like enzymes. They are responsible for the degradation and turnover of cellular [1] and extracellular [2] proteins, covering an essential role in maintaining cell and tissue homeostasis. Different enzymes with endo-, exo-, and endo/exopeptidase activity [3] compose the Cts family. Their proteolytic properties rely on a residue of cysteine in their active site, while other cathepsins are characterized by the presence of aspartic acid or serine amino acid [4]. Cathepsin expression is dysregulated in many pathological conditions, including cancer [5], and their overexpression is traditionally associated with the acquisition of a more aggressive tumor phenotype [6].

Cts, in particular, were shown to play a pivotal role in cancer invasiveness [7], tumor cell communication [8], apoptosis [9], and autophagy [10]. Considering that the Cts family includes 11 different members, only a few of them were tested extensively as tumor pharmacological targets, and, despite the overall scientific opinion, current data regarding the positive or negative contribution of Cts to cancer disease are contradictory [4,11,12,13,14,15,16]. For example, in the lysosomes, they can contribute to the proper function of autophagy, rescuing the cells from exogenous and endogenous stress [17]. In contrast, when they were found free in the cell cytoplasm, they could induce apoptosis through a caspase-independent mechanism of cellular death [9,18]. The role of Cts in cancer disease was shown to depend on their activation state [19] and cellular location [20].

Nevertheless, conclusive progress in the field is hampered by potential redundant and compensatory activities [21] both within the different members of the Cts family [21] and between other cathepsins/proteases [22]. Most of the research performed by far, unveiled the role of single Cts in cancer disease, with few further considerations on how the investigated inhibitors, the genetic silencers, or modifications affected the overall biology of the cells and the expression of the targeted enzymes. These considerations are pivotal to the correct designing of more effective pharmacological interventions and evaluate their long term effects.

**Figure 1:**
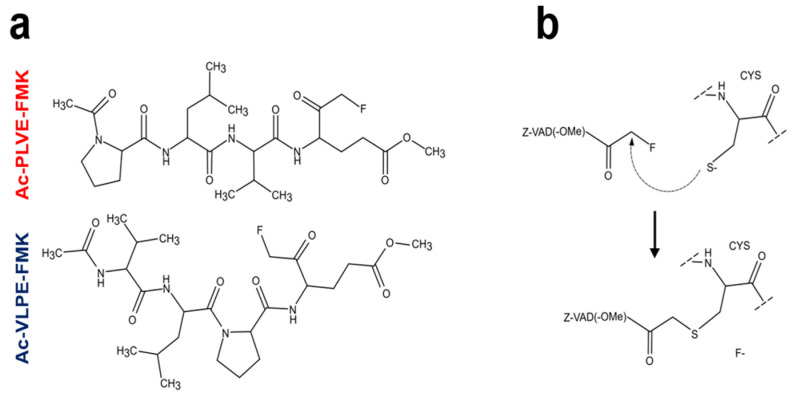
The structure and working mechanism of Ac-PLVE- fluoromethyl ketone (FMK) and Ac-VLPE-FMK inhibitors: (**a**) Inhibitory peptides structure and (**b**) their working mechanism based on Z-VAD-FMK inhibitor.

To address these questions, we designed two small fluoromethyl ketone (FMK)-containing peptides to broadly inhibit the activity against these enzymes. The designing of these inhibitors was inspired by a well-known substrate of the papain-like cysteine protease Triticain-α, derived from *Triticum aestivum* (wheat) [23]. This peptide consists of four amino acids Acetyl-Pro-Leu-Val-Gln (Ac-PLVQ), while the inhibitor sequences are Acetyl-Pro-Leu-Val-Glu-FMK (Ac-PLVE-FMK) and Acetyl-Val-Leu-Pro-Glu-FMK (Ac-VLPE-FMK) (Figure 1a). The working mechanism of both inhibitors should be the same as other selective irreversible cysteine proteinase inhibitors like Z-VAD-FMK (Figure 1b) [24], as well as other FMK-containing drugs. In this class of inhibitors, the FMK group forms a covalent bond with the catalytic cysteine, with the fluorine ion leaving [25,26]. As a model of tumor disease, we chose human renal cancer since this pathology showed aberrant expression of some members of the Cts family [27]. Additionally, further progress in kidney tumor disease depends on the discovery of new targetable markers [28] since when the diagnosis is performed at an advanced stage, the survival rate is low [29], and traditional chemotherapy is ineffective [30]. Investigation on Cts inhibition in this cancer model is rare, and to our knowledge, no comprehensive study in relation to phenotypic and enzymatic alterations has ever been performed. In this work, we tested the biological impact of our inhibitory peptides on the biology of human renal cancer cells, with a focus on the overall lysosomal compartment. We demonstrated that the general inhibition of Cts over longer time periods does not affect the cell proliferation rate. Still, it can affect the overall biology of human renal cancer cells, as well as impacting on the overall Cts expression.

## 2. Results

### 2.1. Computational Modeling of the Peptide Inhibitory Properties on Cts Activity

A docking simulation to predict the interactions of the inhibitors with the binding site of the Cts was performed with the protein-ligand docking software PLANTS [31]. Both Ac-PLVE-FMK and Ac-VLPE-FMK were docked into CtsB, L, and W active sites at pH 4.5, 6.5, and 7.2 since pH can influence the protein interactions through the protonation of the ionizable residues [32]. CtsB and L were chosen as enzymatic models because they have endo and endo/exopeptidase activity, respectively, addressing all the Cts proteolytic mechanisms. They were also shown to play a pivotal role in renal cancer malignancy, and their overexpression was associated with a more aggressive cancer phenotype [33,34,35]. On the other hand, CtsW represents a poorly investigated protease in renal cancer. In contrast, in other investigations, it was shown to preferentially locate in the endothelial reticulum [36], and it is evolutionarily distinguished from CtsB and L [37], representing, therefore, optimal negative control for our research.

**Figure 2:**
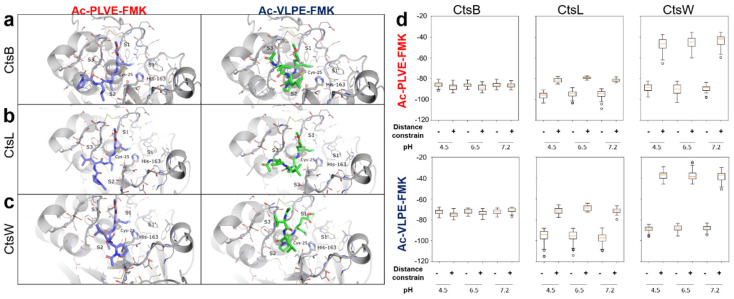
Binding poses of designed inhibitors to cysteine cathepsins (Cts). Docking of the peptides in (**a**) CtsB,(**b**) CtsL, (**c**) CtsW (pH 4.5). Hydrogen atoms are omitted for better depiction. Catalytic Cysteine and Histidine are shown in thick sticks. Inhibitors are shown in purple or green. S1-S3 and S1’ binding pockets are labeled. (**d**) Binding scores (PLANTS Chemplp energy units) for Ac-PLVE-FMK and Ac-VLPE-FMK docking results at different pH levels with and without distance constraints.

Fifty poses per binding site for each ligand were obtained. The analysis revealed that our inhibitors did not bind with the proteases in the pre-reaction state with the fluorine atom of the FMK group in a 3.5 Å radius from the HD2 hydrogen atom of the catalytic histidine and with the carbon atom of the fluoromethyl group in a 3.5 Å radius from the SG atom of the catalytic cysteine. The non-covalent binding energy of the peptides to the proteases is substantially lower than the energy of one covalent bond. Thus, the conformation suitable for covalent bonding may be far from the optimal peptide position in the non-covalent mode. To find non-covalent interactions potentially leading to covalent bond formation, we analyzed the crystal structure of Z-VAD-FMK, covalently bonded to the cysteine protease *Marasmius oreades agglutinin* (PDB id 5D61). In this case, the oxygen atom of the FMK group interacts via hydrogen bonds with the oxyanion hole of the enzyme, formed by the catalytic cysteine backbone N and NE1 atom of Trp-208. For further docking simulations, we added a distance constraint between the oxygen atom of the FMK group of our inhibitors and two hydrogen atoms from the oxyanion holes in the Cts structures. This adjustment allowed for obtaining poses close to the pre-reaction state for both inhibitors. The resulting poses demonstrated that Ac-PLVE-FMK tends to occupy S2 binding sites with either Val or Leu residues, depending on the backbone conformation. The C-terminal Glu residue fitted in the groove around site S1’. However, the N-terminal residue did not bind in S3 or S4 sites and laid closer to the protein surface. Ac-VLPE-FMK instead, tended to bind in S1’-S2′ sites of Cts with its N-terminal residues’ sidechains. Thus, both inhibitors should occupy mostly hydrophobic substrate-binding pockets by Val and Leu side chains. Representative docking of the peptides with the CtsB, L, and W at pH 4.5 is shown in Figure 2a–c. According to the docking score, both inhibitors are less likely to bind in the pre-reaction state, capable of covalent bond formation with CtsW confirming its potential role as a negative control (Figure 2d). Ac-VLPE-FMK also has a weaker binding with all Cts than Ac-PLVE-FMK, possibly due to the lack of conformation variability around Pro residue.

### 2.2. Assessment of Peptide Inhibitory Properties on Cts Activity via Biochemical Assays

Ac-PLVE-FMK and Ac-VLPE-FMK inhibitory properties were evaluated against human recombinant CtsL and B. These recombinant enzymes were expressed in *E. coli* via plasmid transformation and further purification, using nickel-nitrilotriacetic acid (Ni-NTA) sepharose. Gel zymography assay was used to provide a preliminary evaluation of the ability of these recombinant proteins to degrade the gelatin substrate previously embedded in the gel (Supplementary Figure S1). Following Coomassie staining, the Cts were detectable as single bands. As expected, a translucent area was evident, due to the substrate digestion in the proximity of the proteins. Then, the recombinant proteins were tested for their ability to digest the Triticain-α substrate Ac-PLVQ via fluorescent protease activity assay, previously optimized by our group [23]. This probe was conjugated with the fluorogenic chromophore 7-amino-4-methylcoumarin (Ac-PLVQ-AMC). After proteolytic cleavage, it emits a detectable fluorescent signal.

Both human recombinant CtsB and L (20 nM each) were able to cleave the probe Ac-PLVQ-AMC (50 μM) and increase the fluorescent signal detection (red line). In the same experimental conditions, performed in the presence of Ac-PLVE-FMK or Ac-VLPE-FMK (2 μM), the intensity of the fluorescent signal was significantly affected (Supplementary Figure S2—blue and green line, respectively).

**Figure 3:**
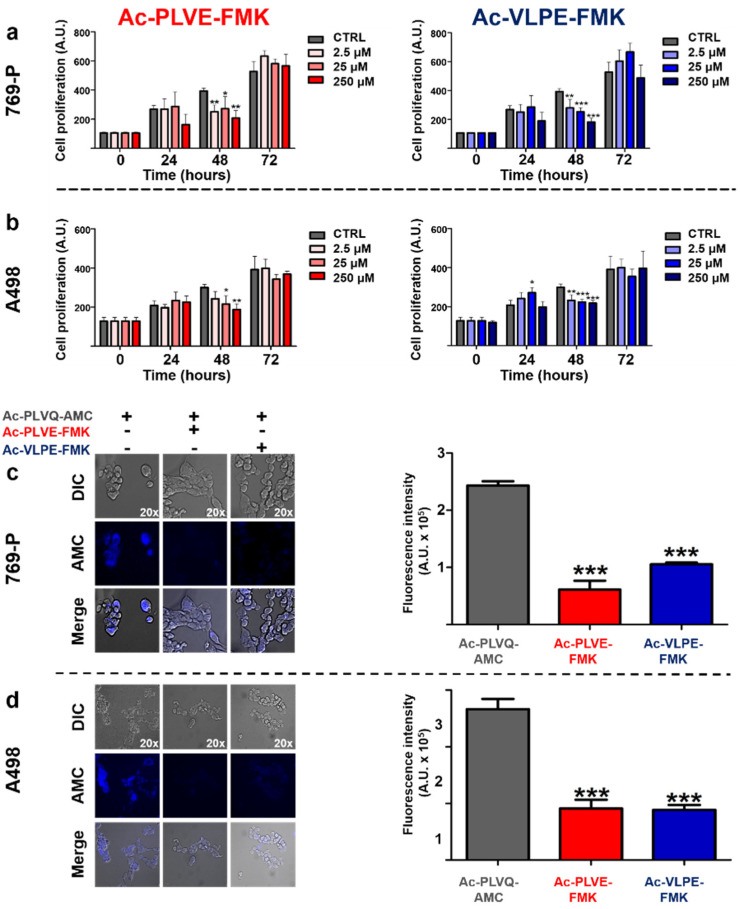
The effect of the peptide inhibitors on human 769-P and A498 renal cancer cell proliferation and proteolytic activity. (**a**) 769-P and (**b**) A498 cells were treated with increasing doses of Ac-PLVE-FMK (red bars) and Ac-VLPE-FMK (blue bars) (2.5–250 μM). Cell proliferation was measured after 24, 48, and 72 h via MTT (3-(4,5-dimethylthiazol-2-yl)-2,5-diphenyltetrazolium bromide) assay. Data represent the mean (±S.D.) of at least three independent experiments, each performed in triplicate. (**c**) Fluorescence microscopy evaluation and quantification of the cell proteolytic activity in 769-P and (**d**) A498 cells towards the fluorogenic substrate Ac-PLVQ-AMC in the absence or presence of Ac-PLVE-FMK and Ac-VLPE-FMK. The cells were seeded in a 96- well plate and after exposure to the peptides for 30 min prior incubation with the fluorescent substrate Ac-PLVQ-AMC for 10 min. Data are expressed as mean (±S.D.), and significance was calculated through one-way ANOVA followed by Dunnet’s test. * = *p* < 0.05, ** = *p* < 0.01, *** = *p* < 0.001.

To evaluate the inhibitory properties of the peptides directly on the human renal cancer cells, we firstly assessed the impact of Ac-PLVE-FMK and Ac-VLPE-FMK on the cell viability of 769-P and A498 cells to determine the working concentrations for further experiments. The peptides were easily dispersed in water and administered at increasing doses to both cell lines for 72 h (Figure 3a,b). The inhibitors showed cytostatic properties on both cell lines only within 48 h of treatment when all the concentrations used negatively impacted on cell proliferation, in particular in the case of 769-P cells. However, after 72 h of incubation, cell viability increased, reaching values not significantly different from the control cells. Further experiments were performed using a concentration of 20 μM. To understand if the peptides could affect the activity of caspase proteases, we tested their effect on cell viability in combination with the chemotherapeutic paclitaxel (PXT) in both the cell lines. The cell line A498 did not result in high mortality even when the cells were treated with PXT alone (data not shown), confirming previously published data [38]. However, in the case of 769-P cells, the ability of the PXT to kill cancer cells was evident after 72 h of treatment at a concentration of 100 nM. When the treatment with PXT was performed in combination with the peptides (20 µM), no significant differences were observed (Supplementary Figure S3). Next, we evaluated the effect of the peptides on renal cancer cell proteolytic activity against the fluorogenic probe Ac-PLVQ-AMC. In this case, 769-p and A498 cells were treated for 30 min with Ac-PLVE-FMK or Ac-VLPE-FMK and were then exposed for 10 min to the substrate Ac-PLVQ-AMC prior fluorescence microscopy analysis (Figure 3c,d). As depicted by the pictures, both the peptides were effective in inhibiting the generation of the fluorescence derived from the cleavage of the substrate in both the cell lines and fluorimetric analysis confirmed that they significantly inhibited the probe degradation. Overall, these data demonstrate that the peptides do not considerably interfere with cell viability and that their inhibitory properties do not affect the proteolytic activity of the proteases that are involved in cell apoptosis; however, they can effectively interfere with the cellular Cts.

### 2.3. Effect of the Inhibitory Peptides on Human Tumor Cell Biology

**Figure 4:**
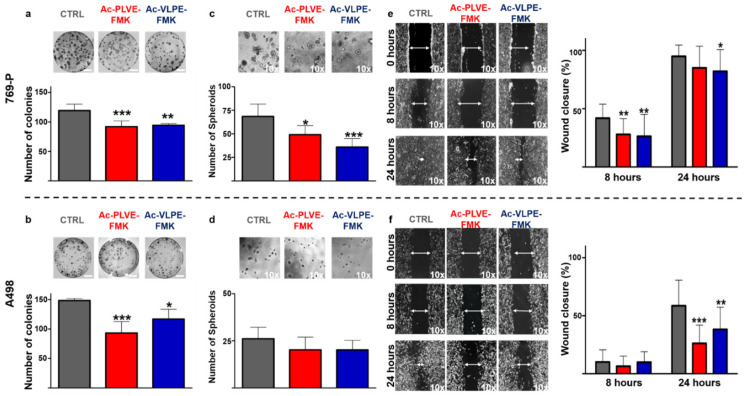
Effect of the peptides on renal cancer cell biology: colony formation assay of (**a**) 769-P and (**b**) A498 under treatment with Ac-PLVE-FMK and Ac-VLPE-FMKI. The cells were treated for 48 h with 20 µM inhibitors and seeded into 10 culture dishes (10 cm diameter) at low confluency where they grew with or without the peptides for an additional 10 days. Representative images of the colony formation assay and quantitative data analysis are shown in the graph. (**c**) 769P and (**d**) A498 spheroid formation evaluated after 7 days of culture on Matrigel coated dishes. The graph is showing the number of spheroids. (**e**) 769-p and (**f**) A498 scratch assay. The graph shows the cell migration rate. All pictures were taken under 10× magnification. Data are expressed as mean (±S.D.), and significance was calculated through one-way ANOVA followed by Dunnet’s test. * = *p* < 0.05, ** = *p* < 0.01, *** = *p* < 0.001.

**Figure 5:**
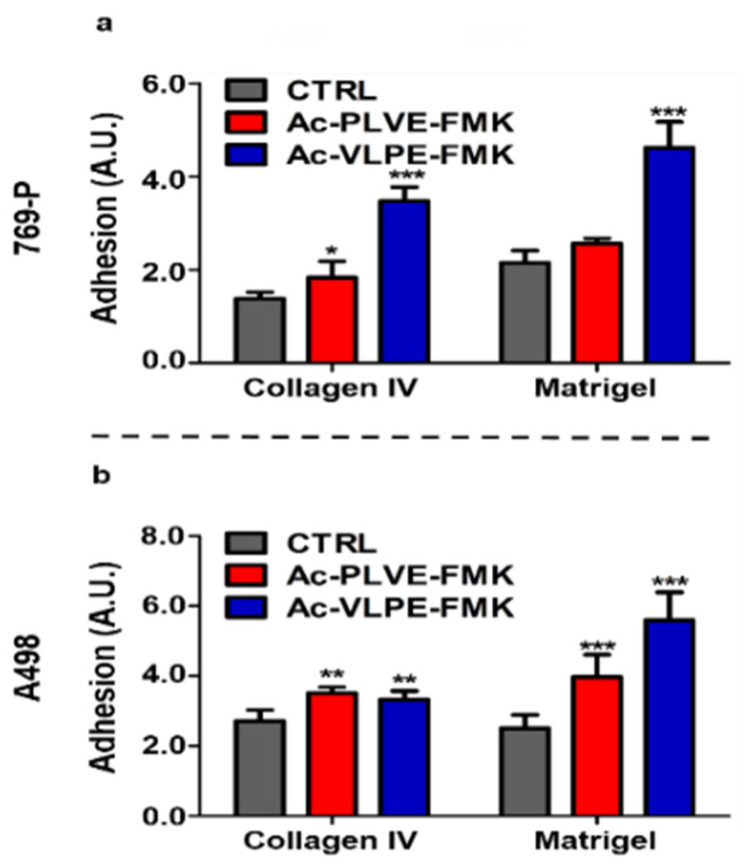
Effect of Cts inhibitors on renal cancer cell adhesion on biological coatings: (**a**) adhesion 769-P and (**b**) A498 cells to collagen IV and Matrigel after treatment with the inhibitors. Data are expressed as mean (±S.D.), and significance was calculated through one-way ANOVA followed by Dunnet’s test. * = *p* < 0.05, ** = *p* < 0.01, *** = *p* < 0.001.

The effective inhibition of specific members of the Cts family was associated with changes in cancer cell properties, such as a decrease in cell invasiveness and migration properties in different tumors, including renal cancer cell lines [39,40,41]. For this reason, various tests were used to evaluate potential changes in cancer cell biology following treatment with the inhibitory peptides. Firstly, we assessed the ability of renal cancer cells to generate colonies. 769-P and A498 cells were treated with the inhibitors for a total of 2 days (20 µM) before being seeded at very low confluency in 10 cm diameter dishes. After cell seeding, the treatment with the peptides was prolonged for an additional 10 days until colony formation was detectable, and identification and quantification of the colonies were performed through Crystal Violet staining. Even though in the case of A498, the colonies were smaller than with 769P cells, both the inhibitors were effective in significantly decreasing colony numbers, as shown in Figure 4a,b. Next, we evaluated the ability of the inhibitory peptides to affect spheroids formation. The cells were treated for 48 h with the Cts inhibitors (20 µM) before being seeded in Matrigel-coated 96-well plates for an additional 7 days. Additionally, in this case, A498 spheroid size and number were smaller than 769-P cells, and the presence of the inhibitors negatively affected the total number of spheroids in both the cell lines (Figure 4c,d), reaching highly significant decreasing values in 769P cells. It is worth noting that, compared to untreated cells, the peptides decreased spheroid size, while increasing their circularity in both the cell lines (Supplementary Figure 4a,b). Finally, we evaluated the ability of the inhibitors to contrast the motility of the cells in a classical scratch healing assay. Human renal cancer cells were seeded and grown until they reached the confluency when a gap was artificially created with a 200 µM tip. As shown in Figure 4e,f, the inhibitors were efficient in a similar fashion in decreasing the gap closure velocity, both at 8 and 24 h after the formation of the scratch. In light of the registered changes in cell phenotype, we hypothesized that the inhibition of Cts could have impacted on cell adhesion to biological substrates. Human renal cancer cells were treated for 48 h before assessing their adhesion properties on collagen IV and Matrigel. Both the peptides induced an increase in cell adhesion on both the biological substrates (Figure 5a,b), and these properties were accompanied by changes in cell stiffness, evaluated through atomic force microscopy analysis (Supplementary Figure S5a,b). Overall, these data demonstrated that the peptides affected renal cancer cell biology.

### 2.4. Effect of the Inhibitory Peptides on E-Cadherin and SNAIL1

**Figure 6:**
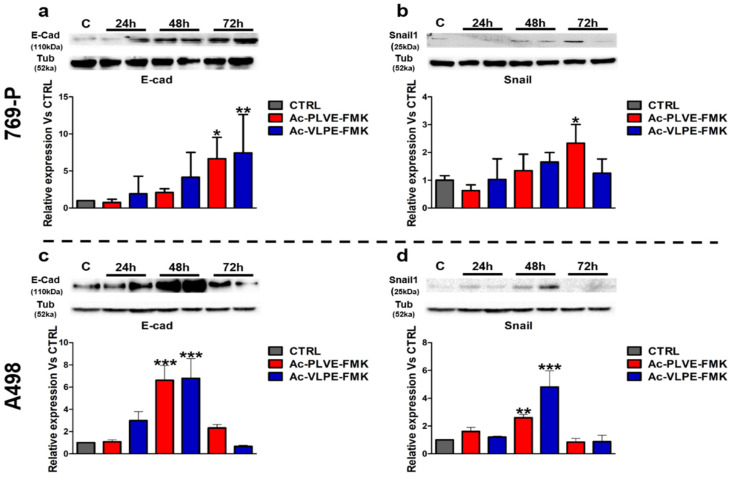
Effects of the peptides on E-cadherin and SNAIL1 protein expression: (**a**,**b**) protein expression of E-cadherin and SNAIL1 in 769-P and (**c**,**d**) A498 cells after 24, 48, and 72 h of treatment with the peptides. Data are represented as mean ± SD of at least three replicates. Significance was calculated through one-way ANOVA, followed by Dunnet’s test.

The collected data indicated that Ac-PLVE-FMK and Ac-VLPE-FMK could affect the overall renal cancer cell phenotype. These properties could be the result of the modulation of the effectors controlling EMT, as previously shown in other tumor models [40,42,43,44]. To test this hypothesis, the cells were treated with the inhibitors for 24, 48, and 72 h by changing the media every day, and they were tested for E-cadherin and SNAIL1 expression (Figure 6; detail information of western blots can be seen in Supplementary Figure S7).

These markers are critical players in the control of EMT, and they are associated with opposite effects on the cancer cell phenotype. While E-cadherin is considered a marker of differentiation, working against EMT (favoring mesenchymal-epithelial transition, as known as MET), SNAIL1 is associated with the acquisition of an undifferentiated phenotype. Both the peptides increased the protein expression of E-cadherin in the first 48 h in both the cell lines (Figure 6). At 72 h, the values of E-cadherin further increased in 769P cells, while decreased in A498 cells. On the other hand, SNAIL1 increased significantly at 72 h only in the case of 769P cells treated with AC-PLVE-FMK, while dropped to values more similar to the control upon treatment with Ac-VLPE-FMK and in A498 cells with both the peptides. More importantly, in both the cell lines, E-cadherin and SNAIL1 followed a similar trend in response to the treatments. Taken together, these data demonstrated that the inhibitory peptides could affect the cell phenotype, involving the genes that control EMT. These phenomena could be at the base of the observed cell adhesion and motility properties, considering that E-cadherin showed the highest increase after treatment, compared to untreated CTRL.

### 2.5. Impact of the Peptides on Lysosomal Biology

**Figure 7:**
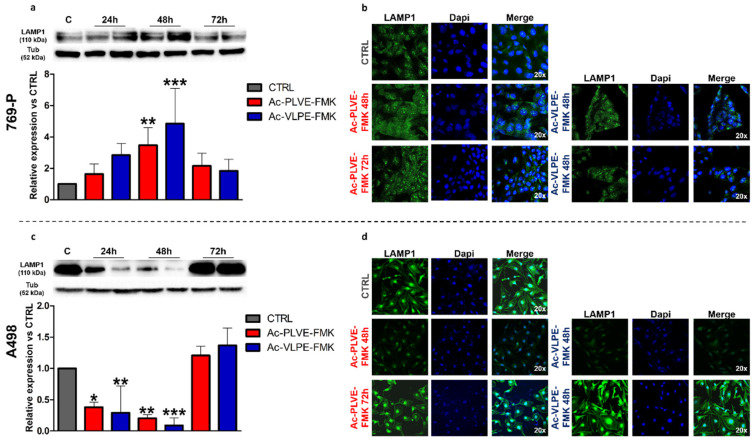
Effect of the peptides on LAMP-1 protein expression: (**a**) Western blotting analysis of LAMP-1 after treatment with PLVE and VLPE (20 μM) for 24, 48, and 72 h in 769-P cells. (**b**) Confocal microscopy evaluation of LAMP-1 expression after 48 and 72 h of treatment with PLVE and VLPE (20 μM) in 769-P cells. (**c**) Western blotting analysis of LAMP-1 after treatment with PLVE and VLPE (20 μM) for 24, 48, and 72 h in A498 cells. (**d**) Confocal microscopy evaluation of LAMP-1 expression after 48 and 72 h of treatment with PLVE and VLPE (20 μM) in A498 cells. Data are represented as mean ± SD of at least three replicates. Significance was calculated through one-way ANOVA followed by Dunnet’s test. * = *p* < 0.05, ** = *p* < 0.01, *** = *p* < 0.001.

To evaluate the potential changes to the lysosomal compartment, we measured the expression of Lysosome Associated Membrane Protein 1 (LAMP-1) over 72 h of treatment. This protein is usually associated with the lysosomal membrane, and it is universally recognized as a marker of these organelles. Western blotting analysis demonstrated a very different effect of the peptides on the modulation of this marker in the two cell lines. In 769-P cells, both the inhibitors significantly increased the expression of this lysosomal biomarker at all the considered time points, reaching significant peaks at 48 h of treatment and generally increasing this marker in all the considered time points. Additionally, Ac-VLPE-FMK demonstrated to be more efficient than Ac-PLVE-FMK in increasing the expression of LAMP-1 (Figure 7a). These data were corroborated by confocal microscopy analysis, confirming that at 48 h, both the peptides increased LAMP-1 expression (Figure 7b), while at 72 h, the content of LAMP-1 decreased towards control levels. On the other hand, LAMP-1 protein expression in A498 cells was significantly affected during the first 48 h, and it was characterized by a substantial recovery at 72 h, reaching values slightly higher than CTRL levels (Figure 7c,d). A similar trend in both the cell lines was observed by analyzing the endolysosomal compartment integrity through Neutral red assay (Supplementary Figure S6) and LysoTracker red fluorescence measurement where we registered slightly increasing and decreasing values only at 48 h of treatment for 769-P and A498 cells, respectively.

**Figure 8:**
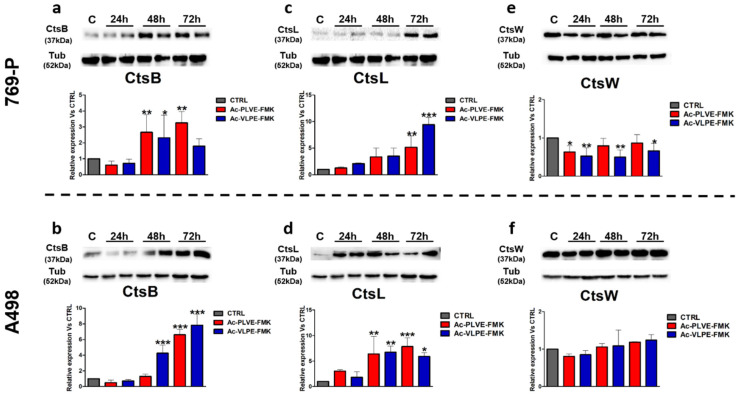
Effect of the peptides on CtsB, L, and W expression: (**a**) protein expression of CtsB in 769-P and (**b**) A498 cells after 24, 48, and 72 h of treatment with Ac-PLVE-FMK and Ac-VLPE-FMK. (**c**) protein expression of CtsL in 769-P and (**d**) A498 cells after 24, 48, and 72 h of treatment with Ac-PLVE-FMK and Ac-VLPE-FMK. (**e**) Protein expression of CtsB in 769-P and (**f**) A498 cells after 24, 48, and 72 h of treatment with Ac-PLVE-FMK and Ac-VLPE-FMK. Data are represented as mean ± S.D. of at least three replicates. Significance was calculated through one-way ANOVA followed by Dunnet’s test.

Next, we evaluated the protein expression of CtsB, L, and W (Figure 8). In both the cell lines, the Cts protein expression was similar. In particular, CtsB and L increased significantly at the later time points of treatment with both the peptides (Figure 8a–d). In contrast, in the case of CtsW, the peptides negatively affected the protein expression in 769-P cells, while no particular differences were detected in A498 cells (Figure 8e,f). Other tested Cts were modulated in their protein expression similarly to CtsB and L. Overall, these experiments indicated that the peptides could affect the biology of the lysosomal compartment and the expression of Cts. The only exception to this rule was represented by CtsW, which did not show a high affinity for the peptides, and its expression was negatively affected by the peptides only in 769-P cells, while no substantial differences were registered in A498 cells.

## 3. Discussion

Cts are universally considered as key players in maintaining proteostasis regulation, and their proteolytic activity is traditionally associated with the lysosomes. However, their sphere of action is not limited just to the lumen of these organelles. They were also detected in the cell cytoplasm [45], nucleus [46], and when secreted, in the extracellular space [46], regulating important processes like autophagy [47], apoptosis [48], gene expression [49], cell signaling [50], and angiogenesis [51]. In this scenario, a potential contribution of Cts in tumor disease is practically obvious, and the first pieces of evidence regarding their role in cancer were published in the late 80s [52]. In the case of renal cancer, the upregulation of CtsB was shown to decrease three- and five-year patient survival rates [33], and CtsK was shown to be overexpressed in renal cell carcinoma patients with Xp11 translocation [53]. Despite detailed data regarding the role of specific Cts in tumor disease, this family of proteases counts 11 members with redundant and compensatory activities, as shown in autoimmune diseases [54] and or thyroglobulin processing [55], respectively. For this reason, strategies aimed at specifically inhibiting the activity of a single Cts member could result in a low impact on cancer cell biology, as well as a limited understanding of the role of these proteases in tumor disease. More importantly, current literature generally did not focus on understanding the impact of the Cts inhibitors on the overall expression of these proteases. In this context, the investigation performed in renal cancer represents an additional novelty since specific studies in the field are very rare in this disease model.

In this work, we designed peptide-based inhibitors that could universally inhibit the action of Cts. Ac-PLVE-FMK and Ac-VLPE-FMK derive from the well-known substrate of the Triticain-α cysteine protease Ac-PLVQ, which was extensively used by our group in previous work to define the activity of this enzyme through fluorimetric assay [23]. The proposed inhibition mechanism is based on FMK-containing drugs, which are known as selective and efficient cysteine protease inhibitors [56]. Molecular docking is a powerful computational approach commonly employed to predict the binding poses and affinities of various ligands to macromolecules. Ligand conformations, obtained in the docking procedure, allow for estimating the interactions required for successful binding and provide insights for further improvements in ligand design. However, this method has limitations, since it does not allow for predicting the occurrence of covalent bonds between macromolecules and ligands [57]. In our case, both Ac-PLVE-FMK and Ac-VLPE-FMK were designed to bind the catalytic cysteine in the Cts covalently; however, the docking model can only estimate the interactions between the peptides and the Cts in the pre-reaction binding.

In the simulation, Ac-PLVE-FMK and Ac-VLPE-FMK tend to occupy the S2 binding site of Cts with aliphatic side chains and form hydrogen bonds by the peptide backbone atoms of the respective residues. However, the N-terminal amino acids of the peptides tend to form fewer contacts with the Cts proteins. Thus, the designed inhibitor molecules can be further improved by the rational design of their N-terminus. According to the docking results, Ac-VLPE-FMK appears to be a weaker binder to all the Cts we considered (see Figure 2d), since the Pro residue hinders the movement of this peptide, thereby reducing the possibility of its proper pre-reaction binding. Thus, Ac-PLVE-FMK theoretically represents a more promising target for further improvement.

Both peptides showed a similar efficiency in inhibiting the activity of recombinant human CtsB and L. More importantly, they demonstrated pronounced inhibitory properties in vitro directly on the cells, implying their ability to penetrate the cell membrane with moderate cytostatic effects only registered during the first 48 h of treatment. Previous work performed with the multi-Cts inhibitor E64 on pancreatic cells showed only a moderated cytotoxic effect, which reached a plateau phase after 48 and 72 h [58]. In general, Cts inhibitors cannot be considered very potent cytostatic molecules. However, as demonstrated in other works, they could increase chemotherapy efficacy [59], even though when used in combination with PXT our peptides did not increase cell toxicity in a significant way. We exclude, however, that the peptides lost their potency over time because the treatments were administered afresh every day. Therefore, we conclude that our peptides do not have a significant impact on renal cancer cell viability.

On the other hand, Cts activity was shown as a modulator of invasive properties, including cellular adhesion [8], anchorage-independent growth [60], colony formation [61], and motility [44] of cells. Our data support this evidence in both the human renal cancer cell lines tested with the peptides, decreasing their ability to migrate in a scratch assay, while increasing their adhesion to biological substrates. It is important to note that differences in cell spreading, associated with more potent cellular adhesive force, can be accompanied by decreased cell migration [62,63]. Additionally, the peptides inhibited colony and spheroid formation, phenomena that can be favored by Cts activity [44,64]. These effects could be a result of an increased E-cadherin expression, a protein involved in cell adhesion, and considered as a marker of differentiation during MET, which increased upon treatment with both the peptides, reaching significant levels at the later time points. A previous work [65] demonstrated that in renal cancer spheroid formation, a down-regulation of E-cadherin occurs, highlighting its potential contribution to the detected anti-spheroid and colony formation properties shown by our peptides.

Interestingly, the inhibitory proteolytic properties of our peptides impacted on Cts homeostasis. We observed different variations in the expression of LAMP-1. At 48 h of treatment with both the peptides, LAMP-1 increased in 769-P cells, while it decreased in A498 cells. However, at 72 h, both the cell lines were characterized by an increase in LAMP-1 expression, even though it did not reach significant levels. These data were corroborated by further fluorescent microscopy analysis as well as by the evaluation of the endosomal compartment integrity performed through neutral red assay and LysoTracker Red. An increase in LAMP-1 expression after CtsB and L knock out was similarly registered in mouse embryonic fibroblast cells [66] and bone marrow-derived macrophages [67].

More importantly, the peptides affected Cts turnover by inducing two different trends in their expression. The CtsB and L expression increased over time after treatment with the peptides in both the cell lines. On the other hand, CtsW protein expression was very stable, and in the case of 769-P cells, it decreased. We can speculate that in the case of CtsB and L, the proteolytic inhibition, induced by the peptides, was counterbalanced by an over-expression of these proteins. This rule was not to apply to CtsW that probably follows other mechanisms of expression regulation [37]. In addition, our data demonstrated a weaker interaction of the inhibitors in the case of the CtsW active site. Compared to other Cts, CtsW was shown to be significantly localized in the endoplasmic reticulum of immune cells [36], and this evidence could form the basis of its differential regulation.

From the pharmacological standpoint, the development of these inhibitors could provide new avenues of research to develop targeted therapies aimed at inhibiting cancer cell proteostasis while impacting their overall phenotype since they showed to affect cell migration and increasing adhesion and expression of E-Cadherin.

Future work is required to take into consideration the potential side effects of this treatment strategy and its impact on cancer biology in vivo, evaluating the peptides’ synergistic effects with current chemotherapeutics, as well as revealing their effects on renal cancer spreading. In addition, more insights are necessary to evaluate their overall effect on the endolysosomal compartment stability, integrity, function (i.e., autophagy) as well on the cell metabolism. On the other hand, the generation of new Cts inhibitors could provide fundamental insights into understanding lysosomal biology and lysosomal-related conditions. In particular, more evidence is necessary to unveil the role of the inhibitors in regulating Cts expression as well as lysosomal turnover, as previously demonstrated by other works [58].

## 4. Materials and Methods

### 4.1. Docking Studies

Crystal structures of CtsB (6AY2) and L (2XU4) were obtained from the PDB databank. The CtsW structure was predicted using Modeller [68,69]. CtsB was used as a template structure. All protein structures were protonated with PROPKA at PDB2PQR server at pH 4.5, 6.5, and 7.2 [69]. Ligand structures were built and optimized in the GAFF force field using Avogadro [70,71].

Docking was performed using PLANTS [72]. The Chemplp scoring function was used in combination with search speed 1. The binding center was set at the SG atom of catalytic cysteine of all considered Cts. Both catalytic cysteine and histidine were set as flexible. When docking with constraints, simple distance constraints between the oxygen atom in the FMK group and atoms H in the catalytic cysteine or amine hydrogens of Gln-23/19/20 in CtsB/L/W were used. The constraint was applied for the distance between 1 and 3 Å. For each ligand, five poses per run were obtained. Ten runs per pH per protein per ligand were made.

### 4.2. Protein Expression and Purification

Total RNA extract from retinoblastoma J79 cells was used to obtain cDNA. A pair of oligonucleotides (TATACATATGCGGAGCAGGCCCTCTTTC and CTCGAGTTAGATCTTTTCCCAGTACTG) was used for the amplification of DNA fragment containing CtsB, the product of which was ligated into pET15b (Merck Millipore, Billerica, MA, USA) using NdeI and XhoI. DNA fragment containing CtsL was amplified using a pair of oligonucleotides (TATAGCTAGCACTCTAACATTTGATCACAGTTT and ATTAAGCTTTCACACAGTGGGGTAGCTG) and ligated into pET28a (+) (Merck Millipore, Billerica, MA, USA) using NheI and HindIII. After the transformation of obtained vectors into Rosetta gammy B(DE3) cells (Merck Millipore, Billerica, MA, USA), these *E. coli* strains were used for the expression of 6His-tagged CathB or CathL using a procedure described by Gorokhovets et al. (2017) for the expression of 6His-tagged papain-like cysteine protease triticain-α [23]. CtsB or CtsL from the insoluble fraction were purified using Ni-NTA sepharose and then refolded using the methods described in detail for protease triticain-α in Gorokhovets et al. (2017).

### 4.3. Gelatin Zymography

A 5× non-reducing loading buffer (0.05% bromophenol blue, 10% SDS, 1.5 M Tris, 50% glycerol) was added to all recombinant proteins: CtsL and B and prior to loading. Then, the proteins were resolved by 12% SDS-polyacrylamide gels containing 0.2% gelatin at 4 °C. Gels were removed, and enzymes were refolded for four washes in 2.5% Triton-X100, 15 min each. Next, the gels were washed twice and incubated in activating buffer (NaAc, pH 4.8, 1 mM EDTA, and 20 mM L-cysteine hydrochloride monohydrate) for 24 h at 37 °C. In the morning, the gels were fixed for 1 h in 50% methanol with 10% acetic acid and then stained for 1 h in Coomassie (10% acetic acid, 25% isopropanol, 4.5% Coomassie Blue). The gels were destained in 10% isopropanol and 10% acetic acid and scanned using the Bio-Rad ChemiDoc MP Imaging System.

### 4.4. Cathepsin Inhibitors

Specific inhibitors for cysteine Cts were developed with the help of computer-graphic modeling, based on the structure of the proteins. The two peptides, Ac-PLVE-FMK and Ac-VLPE-FMK were selected as the specific inhibitors, that provide a binding affinity to Cts and can block their activity. The inhibitors were synthesized by Pepmic (Pepmic Suzhou Jiangsu, China).

### 4.5. Enzymatic Kinetic Studies

The activity of recombinant CtsL and B was detected by the hydrolysis of the fluorogenic substrate Ac-Pro-Leu-Val-Gln- 7-amino-4-methylcoumarin (AMC) (Pepmic Suzhou Jiangsu, China). A total of 20 nM of each protein was mixed in a 96-well plate with 0.1 M sodium acetic buffer (100 mM NaCl, 0.5% DMSO, 0.6 mM EDTA pH 4.6) in the presence or absence of cysteine Cts inhibitor at a final concentration of 2 µM. The substrate was added to a final concentration of 50 μM, and its hydrolysis was continuously measured for 12 min using a CLARIOstar^®^ Plus plate reader (BMG Labtech Ortenberg Baden-Württemberg, Germany) at excitation and emission wavelengths of 353 and 442 nm, respectively.

### 4.6. Cell Culture

The human renal cancer cell line lines 786-P, A498 were obtained from Dr. Vadim Pokrovsky (purchased from American Type Culture Collection). The cells were cultured in RPMI 1640, supplemented with 10% fetal bovine serum and 1% mixture of antibiotics penicillin-streptomycin (all from Gibco, Waltham, MA, USA) at 5% CO_2_ and 37 °C in a humidified chamber. Cells were grown to confluence and harvested by trypsinization, using a 0.25 mg/mL trypsin/EDTA solution (ThermoFisher, Carlsbad, CA, USA) and resuspended in the fresh culture medium. Viable cells were enumerated on the Countess II FL Automated Cell Counter (ThermoFisher, Waltham, MA, USA), following Trypan Blue staining. The cell lines were tested for mycoplasma contamination regularly, using the Molecular Probes™ MycoFluor Mycoplasma Detection Kit (ThermoFisher, Waltham, MA, USA).

### 4.7. MTT

The cell number was evaluated by counting viable cells using the MTT (3-(4,5-dimethylthiazol-2-yl)-2,5-diphenyltetrazolium bromide) colorimetric assay. A total of 2 × 10^4^ cells/well were seeded on independent 96-well plates for each time point (0, 24, 48, and 72 h), with five replicates and treated with two inhibitors (2.5–250 μM). Then, 10 µL of the MTT reagent was added to each well, and cells were incubated for another 5 h. Next, the absorbance value was measured using a CLARIOstar^®^ Plus plate reader (BMG Labtech, Ortenberg, Germany) at 490 nm. Triplicate wells were assayed, and S.D.s were determined.

### 4.8. RNA Extraction and cDNA Synthesis

Total RNA was extracted from cells using the Total RNA isolation kit (Evrogen, Moscow, Russia). Complementary DNA (cDNA) was transcribed from mRNA using a cDNA synthesis kit (Evrogen, Moscow, Russia), according to the manufacturer’s protocols. For RT reaction 1 µg of total RNA was used with optical density OD260/OD280 1.7-2.0 measured with NanoDrop One (ThermoFisher, Waltham, MA, USA).

### 4.9. Western Blot Analysis

Cells were seeded in RPMI containing 10% FBS and cultured for 24 h. Next, 30 µM of inhibitors were added to the culture medium and incubated for 24, 48, and 72 h. Control cells were treated with 0.01% DMSO. At all time points the samples were lysed in 50 mM Tri-HCl (pH 8.0), 100 mM NaCl, 0.5% NP-40, 1% Triton X-100, 1× protease inhibitor cocktail (ThermoFisher, Waltham, MA, USA). The 50 µg of protein lysates were separated by electrophoresis on a 12% SDS-PAGE gel and transferred to the PFDF membranes. The expression of cysteine Cts, LAMP-1, E-cadherin, and SNAIL1 were identified by a reaction with specific primary antibodies (CtsB-Ab190077, Abcam, UK; CtsL-Ab95154, Abcam, UK; CtsW Ab191083; LAMP-1- Ab24170, Abcam, UK; SNAIL1 Ab216347, Abcam, UK and e-cadherin-612131, BD Sciences Franklin Lakes, NJ, USA) which were resuspended in 5% non-fat milk in PBST (all Cts 1:3000, LAMP-1, SNAIL1 and E-cadherin 1:1000) and incubated O/N. The next day, the membranes were washed three times with PBST and incubated for 1 h with secondary antibodies (P-GAR Iss (Goat pAb to rabbit IgG (HRP), Abcam, UK or Rabbit Ab to mouse, Abcam, UK; both 1:5000) in 5% non-fat milk in PBST. After an additional wash (three times with PBS), reactive bands were detected by chemiluminescence (Bio-Rad, Irvine, CA, USA). As a loading control, the membranes were incubated with a polyclonal anti-tubulin antibody (1:5000; Ab52866, Abcam, UK) identically.

### 4.10. Immunofluorescence Staining

The cells were treated for 48 and 72 h, then were fixed in 4% PFA/PBS for 15 min and permeabilized in 0.25% Triton^®^X-100 for 10 min. After blocking the non-specific sites in 2% BSA/PBS-T, the immunofluorescence was performed overnight with primary antibody anti-LAMP-1 (1:100, Abcam, Eugene, OR, USA) incubation, followed by incubation with the appropriate fluorophore-labeled secondary antibody Donkey anti-Rabbit IgG (H+L) ReadyProbes™, Alexa Fluor 488 (1:500; ThermoFisher, USA). The cells were then counterstained with nuclear dye DAPI and visualized under a fluorescent and/or confocal microscope (Olympus BX51, Shinjuku, Tokyo, Japan, and AxioObserver Z1, Zeiss, Oberkochen, Germany) using oil-immersion lenses.

### 4.11. AFM Measurements

Before the AFM experiments, the 50,000 cells were seeded per dish and treated for 48 and 72 h with inhibitors. The AFM measurements were performed at 37 °C, using a commercial atomic force microscope Bioscope Resolve AFM (Bruker, Billerica, MA, USA) combined with an inverted optical microscope (Carl Zeiss, Ulm, Germany). The PeakForce QNM-Live Cell cantilevers (PFQNM-LC-A-CAL, Bruker AFM Probes, USA) with a pre-calibrated spring constant (in a range of 0.06–0.08 N/m) and a 70 nm tip radius was used. The deflection sensitivity (nm/V) was calibrated from the thermal using the pre-calibrated value of the spring constant. The nanomechanical maps were acquired in the force volume mode with a typical map size of 80 × 80 microns and 40 × 40 measurement points [73]. For the force curves, a vertical ramp distance was 3 μm, a vertical piezo speed was 183 μm/s, and the trigger force was 0.5–1 nN. The Young’s modulus (E) was calculated by fitting the force curves with the Hertz model with a bottom-effect correction [73,74].

### 4.12. Scratch Assay

The cells were treated for 48 h with Cts inhibitors in a six-well plate. At experimental time zero, a scratch of culture monolayer was made in each well using a pipette tip. The monolayers were washed with PBS to remove detached cells and cell debris and next refilled with growth medium, including Cts inhibitors. The wells were imaged at time zero and again 6 and 24 h later. Using ImageJ, a measurement was taken for how much the denuded area had filled after 6 and 24 h.

### 4.13. Colony-Forming Assay

769-P and A498 cells were treated 48 h with 30 µM Ac-PLVE-FMK or Ac-VLPE-FMK, next calculated, and 300 cells were placed on 10 cm plates. Cells were maintained in the completed medium with inhibitors for the 10 days, then fixed with 4% paraformaldehyde and stained with 0.4% Crystal Violet solution, finally photographed.

### 4.14. Spheroids Formation Assay

The 769-P cells were treated for 48 h with Cts inhibitors and next suspended in 2% Matrigel in the total medium containing 30 µM Ac-PLVE-FMK or Ac-VLPE-FMK. The 100 prepared cells were seeded in 96-well microplates on top of 50 µL Matrigel (Corning, NY, USA) and incubated for 6 days. The formed spheroids were imaged under an Olympus IX71 microscope, and their number, size, and circularity were measured using ImageJ software. Each experiment had two replicates and was repeated three times.

### 4.15. Adhesion

The 96-well plates were coated with either 15 μg/mL collagen IV (Imtek, Moscow, Russia) or Matrigel 0.5% in RPMI-1640 (Corning, NY, USA) and stored at 4 °C O/N. On the day of the assay, plates were washed twice with PBS and 40,000 cells/well were seeded and incubated for 50 min at 37 °C. Adherent cells were fixed and stained with 0.2% crystal violet/10% ethanol and read at 485 nm on a microplate reader. All the experiments were performed in triplicate.

### 4.16. LysoTracker Red Fluorescence Measurement

First, 1.5 × 10^4^ 769-P or A498 cells were seeded in 96-well plates in full medium. The day after the cells were treated with the peptides for 3 consecutive days to establish 24, 48, and 72 h groups of treatment. LysoTracker Red fluorescence intensity was measured via microplate reader following the protocols described in [75] and applying Ex/Em = 570/600. The cells were incubated with 75 nM of LysoTracker red for 1 h.

### 4.17. Statistical Analysis

All experiments were repeated at least three times. Data are reported as mean ± SD. Data were analyzed using one-way ANOVA, followed by Dunnet’s test (GraphPad, Prism 6.00 for Windows, Graf Pad software, San Diego, CA, USA). The *p*-value of <0.05 was considered statistically significant. with *, similarly *p* <  0.01 with ** and *p *<  0.001 with ***.

## 5. Conclusions

Recently, it has been recognized that the pathogenic function of Cts in cancerogenesis is far more complicated than initially conceived [76]. Experimental studies have shown that many Cts are overexpressed in different tumor types, frustrating every attempt to precisely correlate the role of single Cts with the disease development [77]. In this work, we generated two novel peptides with wide-ranging inhibitory properties towards Cts that could provide new resources to develop new treatments. In particular, they could improve current treatments for conditions such as renal cancer that is resistant to standard chemotherapeutic approaches and could benefit from novel targeted therapies [30,78]. Despite their low cytostatic power, these small inhibitors demonstrated broad inhibiting properties, high membrane permeability, minimal toxicity, and above all, a significant impact on cancer cell phenotype.

Our data demonstrated that the peptides could inhibit Cts activity in two different human renal cancer cell lines impacting their motility, anchorage-independent growth, colony formation, and their adhesion. More importantly, this strategy affected Cts expression, and this evidence should be taken into consideration when similar treatment strategies are designed for cancer and other diseases.

## Supplementary Materials

The following are available online at https://www.mdpi.com/2072-6694/12/5/1310/s1, Figure S1: Detection of recombinant human CtsL and B activity through gelatin zymography assay. Recombinant purified human CtsL and B were loaded on the gelatin-gel zymogram. The Cts were activated through incubation of gel in an activating buffer (pH 4.8). Further staining with Coomassie brilliant blue G-250 showed the induction of proteolytic activity as bright white bands on dark background in correspondence with the bands of the enzymes, Figure S2. Determination of the peptide inhibitory properties against human purified CtsB and L. The activities of human recombinant CtsB and L, were measured via fluorimetric analysis by exploiting the fluorogenic properties of the Triticain-α substrate Ac-PLVQ-AMC. In the assay, the 20 nM of recombinant human CtsL and CtsB were incubated with 50 µM substrate without Cts inhibitors (red line) and with Ac-PLVE-FMK and Ac-VLPE-FMK (blue and green line, respectively) at a concentration of 2 µM. Fluorescence was measured as relative as relative fluorescence unit (RFU), Figure S3. Combined effect of PXT and the inhibitors on 769-P proliferation: 769-P cells were treated with 100 nM of PXT alone or in combination with 20 μM of Ac-PLVE and Ac-VLPE and cell proliferation was measured after 24, 48, and 72 h via MTT assay. Data represent the mean (± S.D.) of three independent experiments, each performed in triplicate, Figure S4. Spheroids size and circularity: (**a**,**b**) size and circularity of 769-P and (**c**,**d**) A498 cells upon treatment with Ac-PLVE-FMK and Ac-VLPE-FMK. Data represent the mean (±S.D.) of three independent experiments, each performed in triplicate, Figure S5. Effect of Cts inhibitory peptides on cell stiffness: representative optical phase contrast image of cells (first row) and stiffness maps determined by indentation (Young’s modulus) of 769-P cells (second row) with and without treatment with PLVE and VLPE. Calibration bars represent 25 μm. The graph shows the values of Young’s modulus of all analyzed samples, data are expressed as mean (± S.D.) and significance was calculated through one-way ANOVA followed by Dunnet’s test. * = *p* < 0.05, ** = *p* < 0.01, Figure S6. The effect of the peptide inhibitors on human 769-P and A498 renal cancer cell on lysosomes integrity. (**a**) 769-P and (**b**) A498 cells were treated with increasing doses of Ac-PLVE-FMK (red bars) and Ac-VLPE-FMK (blue bars) (2.5–250 μM). NR uptake was measured after 24, 48, and 72 h via MTT assay. (**c**) LysoTracker red evaluation in 769-P and (**d**) A498 cells after 24, 48, and 72 h with the inhibitors (20 μM). Data represent the mean (± S.D.) of at least three independent experiments, each performed in triplicate, Figure S7. Uncropped western blots.
