# Supplementary material for: Cysteine Cathepsins Inhibition Affects Their Expression and Human Renal Cancer Cell Phenotype
Source: Cancers (Basel). 2020 May 21;12(5):1310. doi: 10.3390/cancers12051310 (PMC7281206; doi:10.3390/cancers12051310)
Supplement: Supplementary file 1 [file cancers-12-01310-s001.pdf]

Cysteine Cathepsins Inhibition Affects Their Expression and Human Renal Cancer Cell Phenotype

Magdalena Rudzińska, Alessandro Parodi, Valentina D. Maslova, Yuri M. Efremov, Neonila V. Gorokhovets, Vladimir A. Makarov, Vasily A. Popkov, Andrey V. Golovin, Evgeni Y. Zernii [1]and Andrey A. Zamyatnin, Jr.

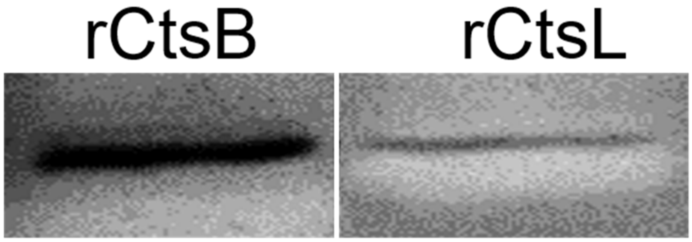

Figure S1. Detection of recombinant human Cts L and B activity through gelatin zymography assay. Recombinant purified human Cts L and B were loaded on the gelatin-gel zymogram. The Cts were activated through incubation of gel in an activating buffer (pH 4.8). Further staining with Coomassie brilliant blue G-250 showed the induction of proteolytic activity as bright white bands on dark background in correspondence with the bands of the enzymes.

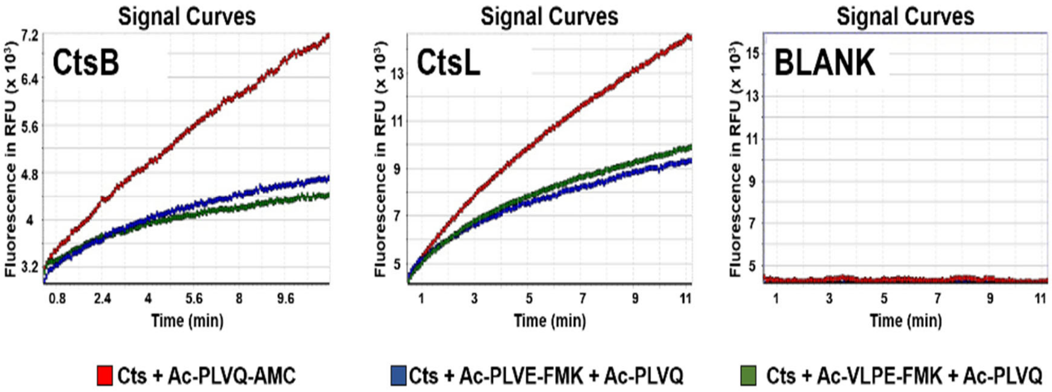

Figure S2. Determination of the peptide inhibitory properties against human purified CtsB and L. The activities of human recombinant Cts B and L, were measured via fluorimetric analysis by exploiting the fluorogenic properties of the Triticain-α substrate Ac-PLVQ-AMC. In the assay, the 20 nM of recombinant human Cts L and Cts B were incubated with 50 μM substrate without Cts inhibitors (red line) and with Ac-PLVE-FMK and Ac-VLPE-FMK (blue and green line, respectively) at a concentration of 2 μM. Fluorescence was measured as relative as relative fluorescence unit (RFU).

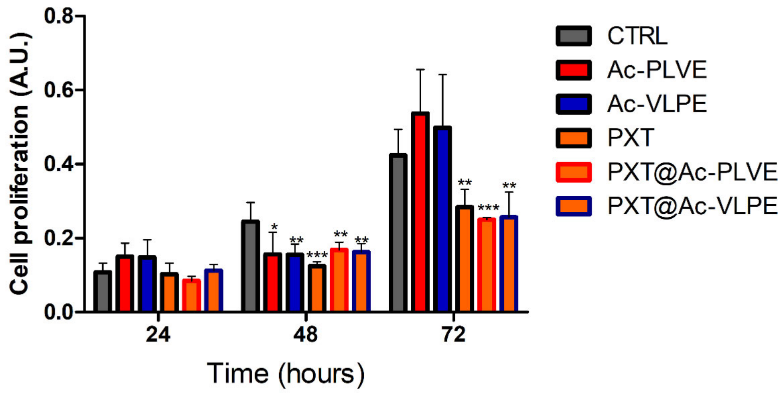

Figure S3. Combined effect of PXT and the inhibitors on 769-P proliferation: 769-P cells were treated with 100 nM of PXT alone or in combination with 20 μM of Ac-PLVE and Ac-VLPE and cell proliferation was measured after 24, 48, and 72 h via MTT assay. Data represent the mean (± S.D.) of three independent experiments, each performed in triplicate.

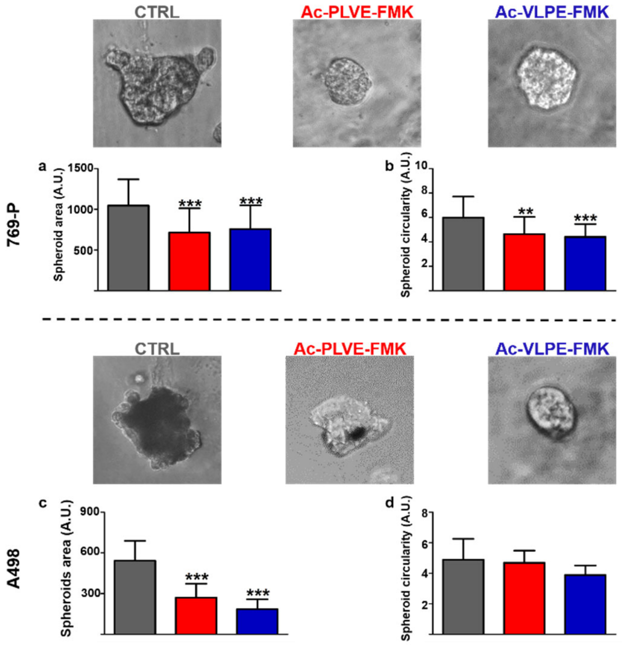

Figure S4. Spheroids size and circularity: (**a**,**b**) size and circularity of 769-P and (**c**,**d**) A498 cells upon treatment with Ac-PLVE-FMK and Ac-VLPE-FMK. Data represent the mean (± S.D.) of three independent experiments, each performed in triplicate.

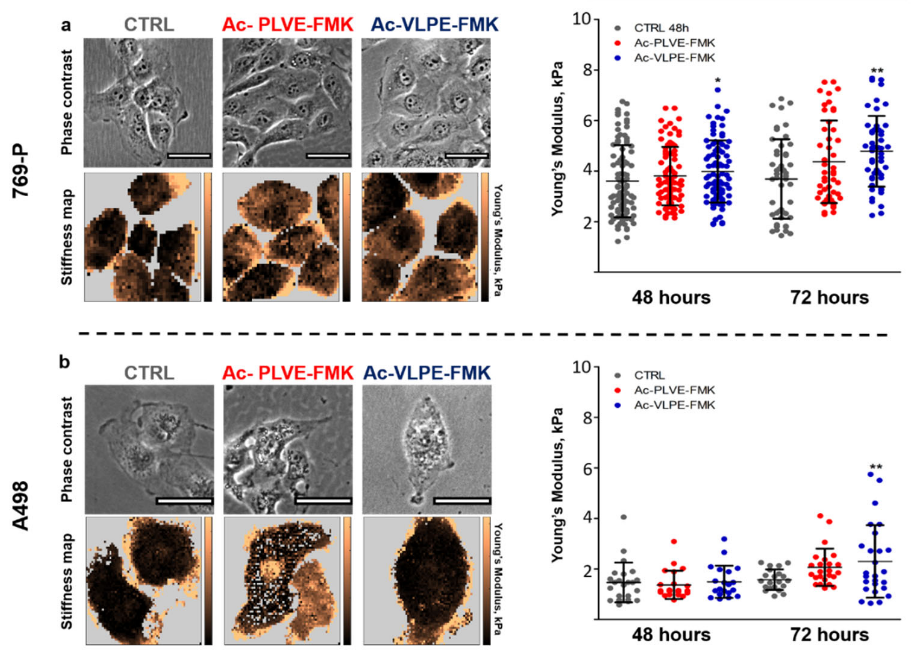

Figure S5. Effect of Cts inhibitory peptides on cell stiffness: representative optical phase contrast image of cells (first row) and stiffness maps determined by indentation (Young's modulus) of 769-P cells (second row) with and without treatment with PLVE and VLPE. Calibration bars represent 25 µm. The graph shows the values of Young's modulus of all analyzed samples, data are expressed as mean (± S.D.) and significance was calculated through one-way ANOVA followed by Dunnet's test. * = $p < 0.05$, ** = $p < 0.01$.

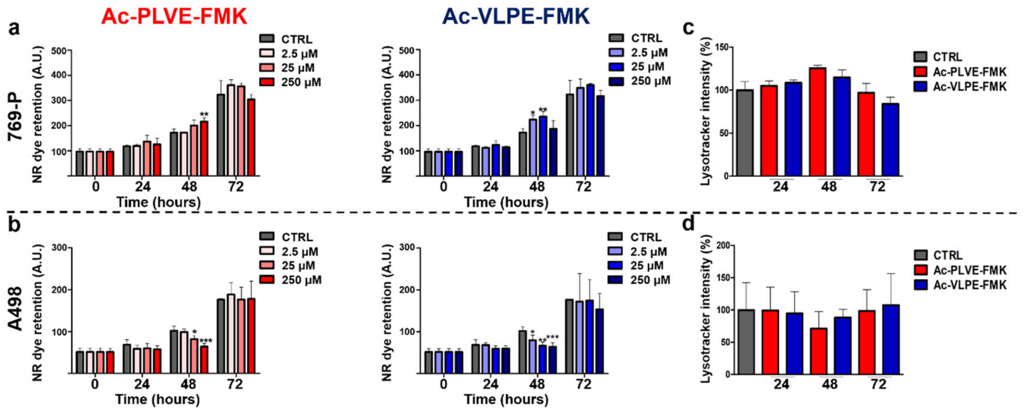

Figure S6. The effect of the peptide inhibitors on human 769-P and A498 renal cancer cell on lysosomes integrity. (**a**) 769-P and (**b**) A498 cells were treated with increasing doses of Ac-PLVE-FMK (red bars) and Ac-VLPE-FMK (blue bars) (2.5–250 µM). NR uptake was measured after 24, 48, and 72 h via MTT assay. (**c**) LysoTracker red evaluation in 769-P and (**d**) A498 cells after 24, 48, and 72 h with the inhibitors (20 µM). Data represent the mean (± S.D.) of at least three independent experiments, each performed in triplicate.

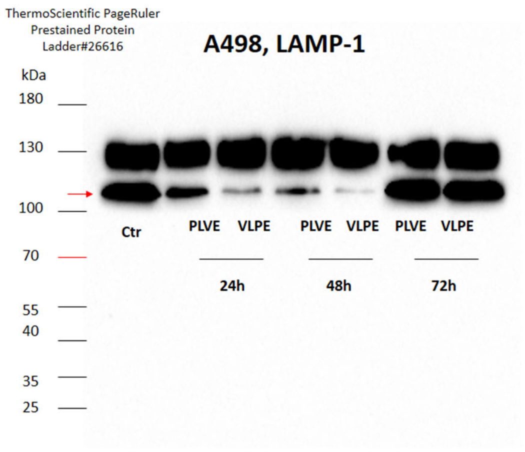

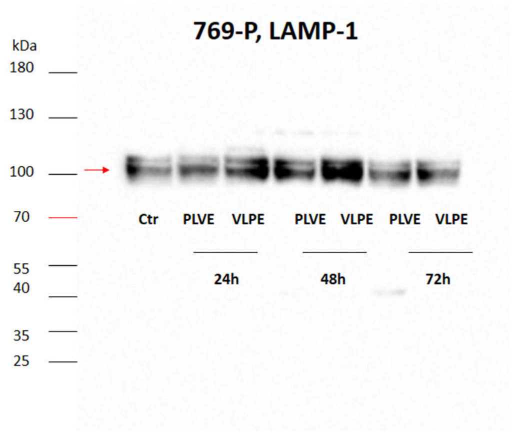

* The mean, normalized densitometry measurements for all samples (each protein) are provided in the main text of the paper.

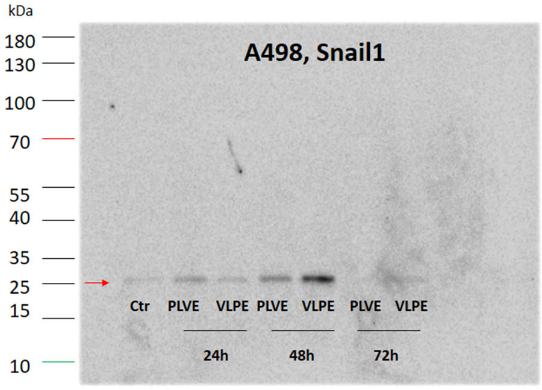

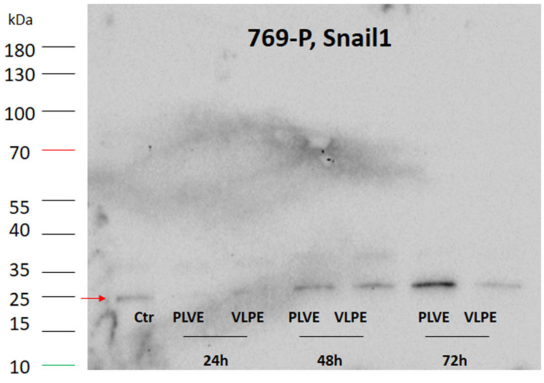

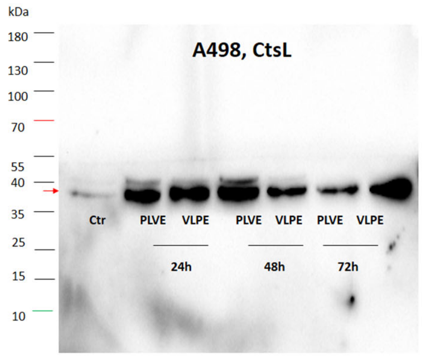
A498, CtsL
kDa
180
130
100
70
55
40
35
25
15
10
Ctr
PLVE
VLPE
PLVE
VLPE
PLVE
VLPE
24h
48h
72h

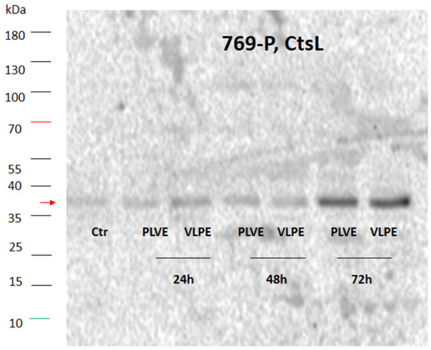
769-P, CtsL
kDa
180
130
100
70
55
40
35
25
15
10
Ctr
PLVE
VLPE
PLVE
VLPE
PLVE
VLPE
24h
48h
72h

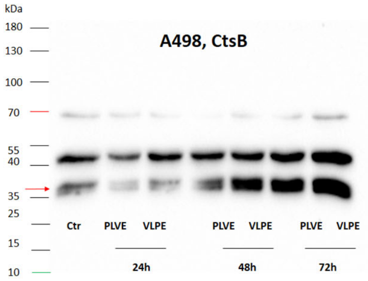
A498, CtsB
kDa
180
130
100
70
55
40
35
25
15
10
Ctr
PLVE
VLPE
PLVE
VLPE
PLVE
VLPE
24h
48h
72h

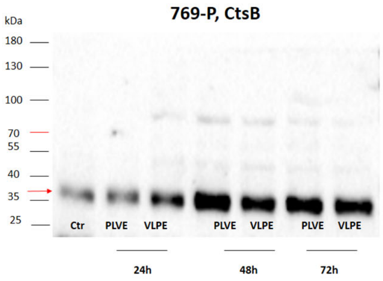
769-P, CtsB
kDa
180
130
100
70
55
40
35
25
Ctr
PLVE
VLPE
PLVE
VLPE
PLVE
VLPE
24h
48h
72h

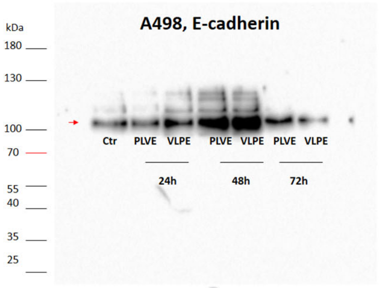
A498, E-cadherin
kDa
180
130
100
70
55
40
35
25
Ctr
PLVE
VLPE
PLVE
VLPE
PLVE
VLPE
24h
48h
72h

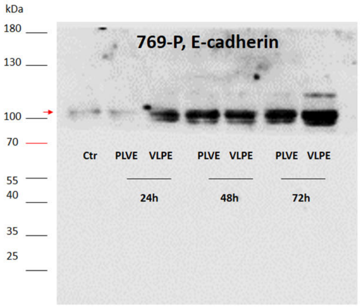
769-P, E-cadherin
kDa
180
130
100
70
55
40
35
25
Ctr
PLVE
VLPE
PLVE
VLPE
PLVE
VLPE
24h
48h
72h

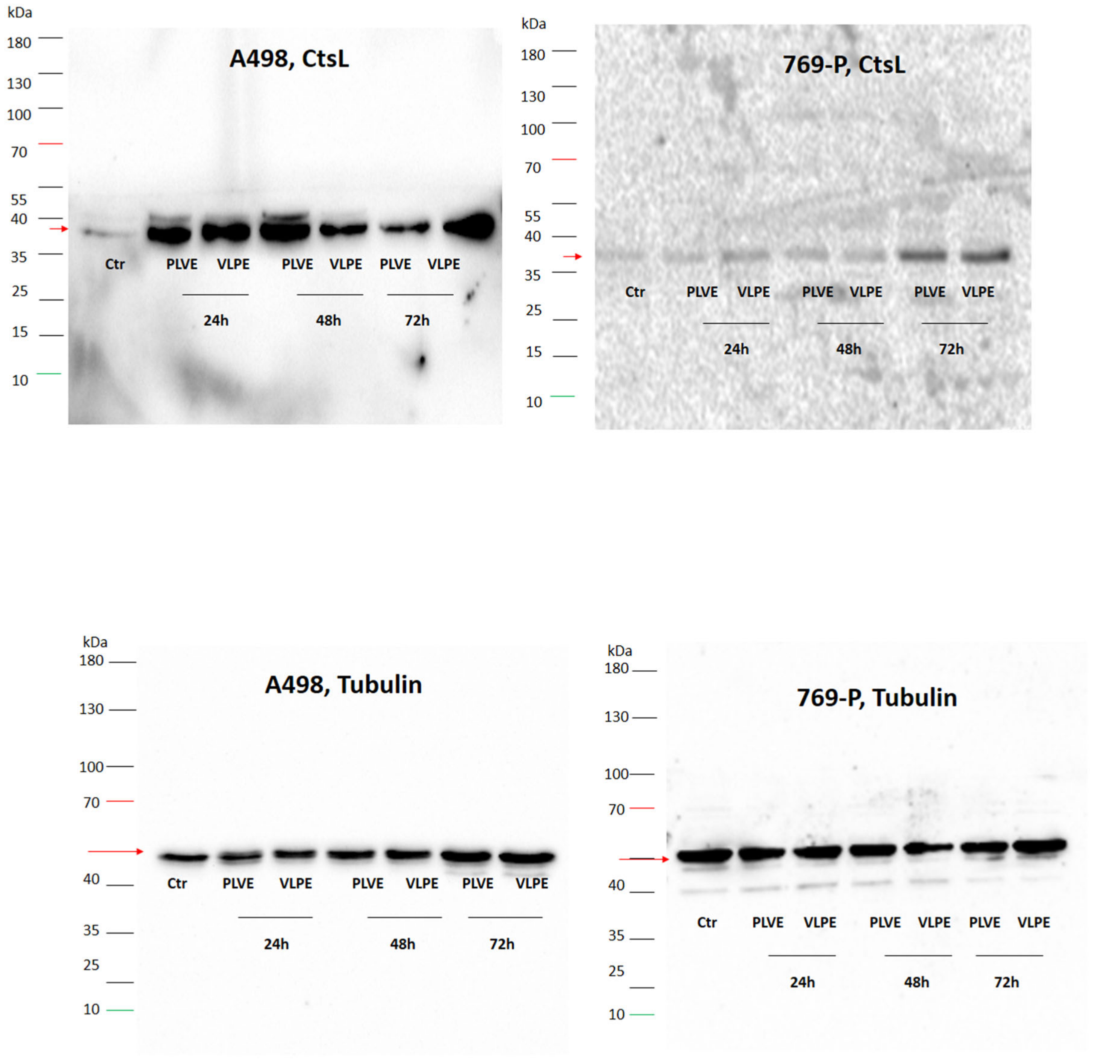

Figure S7. Uncropped western blots.
